# AI: Bridging Ancient Wisdom and Modern Innovation in Traditional Chinese Medicine

**DOI:** 10.2196/58491

**Published:** 2024-06-28

**Authors:** Linken Lu, Tangsheng Lu, Chunyu Tian, Xiujun Zhang

**Affiliations:** 1 North China University of Science and Technology Tangshan China; 2 National Institute on Drug Dependence and Beijing Key Laboratory of Drug Dependence Research Peking University Beijing China; 3 School of Psychology and Mental Health North China University of Science and Technology Hebei Province China

**Keywords:** traditional Chinese medicine, TCM, artificial intelligence, AI, diagnosis

## Abstract

The pursuit of groundbreaking health care innovations has led to the convergence of artificial intelligence (AI) and traditional Chinese medicine (TCM), thus marking a new frontier that demonstrates the promise of combining the advantages of ancient healing practices with cutting-edge advancements in modern technology. TCM, which is a holistic medical system with >2000 years of empirical support, uses unique diagnostic methods such as inspection, auscultation and olfaction, inquiry, and palpation. AI is the simulation of human intelligence processes by machines, especially via computer systems. TCM is experience oriented, holistic, and subjective, and its combination with AI has beneficial effects, which presumably arises from the perspectives of diagnostic accuracy, treatment efficacy, and prognostic veracity. The role of AI in TCM is highlighted by its use in diagnostics, with machine learning enhancing the precision of treatment through complex pattern recognition. This is exemplified by the greater accuracy of TCM syndrome differentiation via tongue images that are analyzed by AI. However, integrating AI into TCM also presents multifaceted challenges, such as data quality and ethical issues; thus, a unified strategy, such as the use of standardized data sets, is required to improve AI understanding and application of TCM principles. The evolution of TCM through the integration of AI is a key factor for elucidating new horizons in health care. As research continues to evolve, it is imperative that technologists and TCM practitioners collaborate to drive innovative solutions that push the boundaries of medical science and honor the profound legacy of TCM. We can chart a future course wherein AI-augmented TCM practices contribute to more systematic, effective, and accessible health care systems for all individuals.

## Introduction

Traditional Chinese medicine (TCM) is a vibrant and enduring medical system that has been refined for thousands of years, thus offering a rich tapestry of health and healing practices [1]. With its roots deeply embedded in Chinese philosophy and a profound understanding of the human body’s relationship with the natural world, TCM has developed a unique set of diagnostic and therapeutic methodologies. These methodologies include herbal medicine, acupuncture, and the identification of syndrome patterns, which are grounded in the fundamental concepts of yin and yang and the 5-element theory, thus providing a holistic approach to health that addresses the body, mind, and spirit. As a traditional medical system, TCM primarily depends on personal experience and lacks standardized and systematic diagnosis and treatment procedures, which potentially discourages its more widespread adoption. Thus, the rapid expansion of artificial intelligence (AI) can significantly improve the reliability and accuracy of TCM diagnostics, thereby increasing the effective use of therapeutic methods for patients [2]. AI is currently being used in diagnostics to detect abnormalities in medical imaging, such as identifying lung nodules or other suspicious lesions in early cancer screening [3]. These tools can assist physicians in making an initial diagnosis by analyzing large amounts of imaging data to identify potential signs of cancer and can also help physicians more accurately characterize suspicious lesions, including their shape, volume, histopathological diagnosis, disease stage, and molecular features. AI also has potential therapeutic value for the diagnosis of mental disorders such as major depressive disorder by successfully distinguishing patients from healthy controls through machine learning [4,5]. This characterization is critical for determining treatment options and predicting disease progression. However, applying AI to TCM exhibits a range of both opportunities and challenges. AI can enhance the diagnostic process by enabling physicians to make more accurate assessments of diseases and patient constitutions through the analysis of extensive TCM clinical data. It can also be instrumental in creating personalized treatment plans that are tailored to the unique conditions of each patient. Concurrently, the integration of AI into TCM raises significant concerns about patient privacy and data security. Although the establishment and learning of classification criteria still cannot eliminate subjectivity, efforts must be made to refine these processes. Addressing these issues necessitates the establishment of stringent ethical standards and robust privacy measures.

The successful integration of AI into TCM is contingent upon collaborative efforts across various disciplines, including medicine, computer science, and data science. Building interdisciplinary teams and fostering effective communication are crucial for driving innovation in this field. As technology evolves, it possesses the potential to revolutionize TCM practices provided that it is implemented with careful consideration of the ethical implications and needs of the TCM community. In the realm of TCM, diagnosis is a pivotal element wherein practitioners engage in comprehensive inquiry and conduct head-to-toe examinations of patients. This process is central to collecting health-related data. Such a diagnostic approach facilitates an in-depth evaluation of the patient’s overall health status coupled with a nuanced understanding of the fundamental characteristics of the disease. TCM diagnosis is based on a holistic perspective and involves a comprehensive assessment of both physiological and psychological aspects of health rather than relying solely on objective diagnostic criteria such as molecular markers and physiological indicators. There are generally 4 main diagnostic methods in TCM: inspection, auscultation and olfaction, inquiry, and palpation. These facets of TCM have been widely accepted by TCM practitioners worldwide. This aspect of TCM is particularly pronounced for highly skilled physicians who are able to derive diagnostic insights through visual inspection alone, often without an explicit need for detailed procedural explication. Moreover, they believe that nonrational thinking, which encompasses implicit meanings, intuition, inspiration, and imagination, plays a vital role in TCM diagnosis and treatment [6]. In conjunction with the concept of the 4 diagnostic methods, practitioners assess and integrate various clinical data to investigate evidence, identify a disease’s root causes, establish treatment strategies, assess treatment efficacy, and anticipate healing progress [7]. The integration of AI into TCM diagnosis respects and uses the intuition and experience of practitioners, thus serving as an auxiliary means of clinical research to evaluate and verify diagnostic results rather than replace human judgment. AI can be designed to analyze complex patterns in patient data, thereby augmenting traditional diagnostic methods. For example, machine learning models can be trained to recognize subtleties in patient inquiry responses, thus enhancing the practitioner’s ability to identify the root causes of diseases and tailor treatment strategies. AI systems can be developed to consider the holistic approach that is inherent in TCM. By analyzing a wide range of clinical data, AI can provide a more comprehensive health assessment that aligns with the TCM concept of integrating various aspects of a patient’s condition. This not only supports the practitioner’s diagnostic process but also aids in anticipating healing progress and the efficacy of treatments. In addition, AI can tailor treatments to patients’ individual needs and conditions by considering their unique body states and responses to various therapeutic interventions, thus leveraging the ability of AI to process and learn from large data sets as well as the enormous potential for personalized treatment in TCM. The role of AI in TCM is to augment the expertise of physicians, thus providing insights and analytics that support the holistic and personalized approach to health care that is at the heart of TCM. By doing so, AI can contribute to enhancing patient care and ensuring that the rich heritage of TCM is carried forward and developed in the modern health care era.

## AI in TCM Diagnosis

### Inspection

In the practice of TCM, the method of inspection primarily focuses on the acquisition of diagnostic information through direct observation. This approach involves assessing the patient’s condition by scrutinizing various physical changes across the body. Distinct from the paradigm of Western medicine, which predominantly relies on objective, empirical evidence, TCM tends to base its diagnostic conclusions on subjective interpretations by medical practitioners [8]. The scope of inspection for diagnosis is quite broad. Although the methods comprise craniofacial observations, tongue and face diagnoses are the primary methods used for inspection. An inspection of the tongue’s shape, size, color, and texture aids in the assessment of organ function and the development of medical conditions. Facial expression analysis is a diagnostic method that aligns with the theory of 5 zang organs, corresponding to 5 elements and colors. It involves distinguishing different changes in facial color, such as green, red, yellow, white, and black, based on the principles of yin and yang and the 5-element theory [9]. Building on this traditional foundation, technological advancements have introduced new methods to enhance TCM diagnostics. For example, the development by Chen [10] of a neural network–based system marked a significant advancement in this research. This innovative platform automates the diagnostic and treatment process in TCM with a focus on symptom analysis. It empowers physicians to efficiently access crucial medical records, thus providing valuable insights into the effectiveness of TCM treatments for similar conditions. Furthermore, the system streamlines the prescription process, thus enabling precise electronic prescriptions to be quickly dispatched to relevant departments and patients. This integration of AI with TCM improves diagnostic accuracy and facilitates the sharing of expertise among practitioners, thus ultimately enhancing the standard of care in TCM. AI is currently one of the most discussed topics in medical imaging research. It serves as a significant enabler for handling vast amounts of medical images, thereby deciphering disease features that may be imperceptible to the human eye. Similarly, AI-based facial diagnosis and tongue diagnosis hold promise for further development. Recently, Liu et al [11] reviewed AI methods in the field of tongue diagnosis. They identified two main challenges that hinder development in this field: (1) the authority of data sets and (2) a misconception about a sole reliance on single features for diagnosis in traditional Chinese tongue diagnosis [11]. The combination of AI with this field overcomes the inherent subjectivity of TCM diagnosis and provides a more objective and standardized approach to tongue diagnosis. Technological advances such as multiscale features and the incorporation of previous knowledge have been successfully applied to improve the accuracy and reliability of AI-assisted tongue analysis. In addition, robust data sets and reliable performance evaluations are still needed to address existing problems in the field. The future of intelligent tongue diagnosis is promising, with potential breakthroughs in self-supervised methods, multimodal information fusion, and tongue pathology research that are expected to have a significant impact on research and clinical practice. On the basis of this scenario, we propose potential solutions to address these issues. First, standardizing data sets for tongue diagnosis should be a collaborative effort that involves experts in TCM. Second, leveraging multimodal data in AI is a crucial approach for the AI-driven transformation of TCM.

The future of AI in the inspection component of TCM is poised to transform traditional diagnostic practices through innovative research and practical applications. One of the primary research directions is the development of sophisticated AI algorithms that can analyze and interpret tongue and facial diagnostics at a level of detail that surpasses human perception. By training these algorithms on diverse and high-quality data sets, AI systems can learn to identify subtle patterns and changes that indicate underlying health conditions, thus complementing the expertise of TCM practitioners.

### Auscultation and Olfaction

Auscultation and olfaction in TCM involve the use of a physician’s hearing to detect changes in a patient’s voice and sounds. Olfaction relies on the physician’s sense of smell to detect changes in odors. The theoretical basis for these practices in TCM is the belief that a patient’s speech sounds and body odors can reflect the physiological and psychological states of their internal organs. Consequently, auscultation and olfaction have long been highly regarded in the field of TCM. However, objective studies and literature on auscultation and olfaction are scarce, which may be attributed to the complex acoustic properties of sounds, including a plethora of natural noises, similar acoustic signals, and diverse chemical compositions of thousands of volatile organic compounds in exhaled gases. These factors have hindered the development of objective research on TCM auscultation and olfaction. Chiu et al [12] introduced quantifiable parameters for TCM auscultation, which allowed for the identification of nonvacuity, qi vacuity, and yin vacuity characteristics in participants. This quantification process enhances the practice of TCM auscultation. There is still a need for more quantitative data on auscultation and further advancements in the application of AI to analyze such quantitative data. The integration of AI into this method is facilitated through the use of advanced sensor technologies and audio analysis algorithms. For example, digital stethoscopes can capture and record bodily sounds with greater clarity. These sounds are then processed by AI algorithms that can filter out background noise and enhance the relevant audio signals. With regard to objective olfactory analysis, there have been several recent studies from a TCM perspective. A recent study introduced a new odor map with the ability to characterize odor quality that was comparable to that of highly skilled human “sniffers” [13]. The algorithms of these odor detection tools have significant potential for quantifying olfactory diagnosis in TCM. AI algorithms are then applied to data generated by these devices to identify specific volatile organic compound profiles that are associated with different health conditions. This is a complex task given the vast number of potential volatile organic compounds and their concentrations; however, machine learning models have shown the ability to handle this complexity and provide objective data for diagnosis. Therefore, the primary focus should be on building an AI odor monitoring system. Such a detection system can be developed by selecting specific biomarker reagents [14]. The development of diagnostic molecular biomarkers for olfaction diagnosis in TCM is also a substantial task. These biomarkers should be capable of quantifying the olfactory diagnostic process in TCM more accurately.

However, there are still some challenges in the application of AI to auscultation and olfaction, including challenges regarding data quality and standardization. The collection of auditory and olfactory data requires highly accurate sensors and devices. Data quality and standardization are critical for training accurate AI models. Inaccurate or inconsistent data can lead to misjudgments by the AI system. The second challenge involves the recognition of extremely complex sound and smell patterns that can be perceived differently among individuals. AI needs to be able to recognize and understand these complexities, which places high demands on the design and training of algorithms. We need to explore and develop more advanced sensor technologies to improve the accuracy and consistency of data collection and use deep learning techniques to improve the ability of AI models to recognize complex sound and odor patterns.

### Inquiry

Interrogation diagnosis (or inquiry diagnosis) directly asks patients questions about various physiological and psychological feelings. This methodology includes gathering information about the patient’s family history, primary complaints, living conditions, dietary habits, sleep patterns, and other physical condition characteristics. This process allows the practitioner to gain a comprehensive understanding of the patient’s overall health, including factors that may contribute to their current condition. A thorough understanding of a patient can also avoid the influence of a previous medical history on treatment. The inquiry aims to provide a holistic view of the patient in consideration of not only physical symptoms but also lifestyle and environmental factors that could impact their health. The content of TCM inquiries is mainly based on the “Ten Brief Inquiries”; however, at present, TCM inquiries also incorporate past history, allergy history, and family history from modern medical records [15]. The GatorTron system, which was developed by Yang et al [16], enhances the use of clinical narratives in the creation of various medical AI systems, thus ultimately leading to better health care delivery and health outcomes. However, electronic health record (EHR) analysis for TCM inquiry is not yet well developed and primarily relies on natural language extraction techniques to extract electronic medical record data, which are then used to establish a knowledge repository for traditional Chinese clinical cases. For example, AI systems are capable of identifying TCM-specific symptoms such as “fatigue” and “dry mouth” from patient narratives, thus correlating these symptoms with associated internal organ imbalances. This sophisticated recognition aids physicians in assessing patients’ constitutions and developing personalized treatment plans. For individuals with chronic conditions, AI facilitates a more in-depth analysis by sifting through extensive health records to forecast disease progression, thereby providing physicians with a solid foundation for accurate diagnoses. Moreover, AI extends its support to patients who require ongoing care by offering tailored advice on diet and exercise, thus significantly contributing to the enhancement of their quality of life and the mitigation of relapse risks. The advent of smart wearables has further empowered AI by enabling real-time health data collection, which is swiftly relayed to AI for analysis. This system proactively notifies health care providers and patients about emerging health concerns, thus exemplifying the potential of AI in diagnostics and proactive patient care within the framework of TCM.

In the future, it will be essential to confirm the accuracy of large language models (LLMs) such as GPT-3.5 and GPT-4 in TCM diagnosis [17]. This process requires a nuanced approach that acknowledges the complexity and richness of TCM terminology. The first step is to collect comprehensive patient data, including symptoms, medical history, lifestyle factors, and any other relevant information. These data must be preprocessed to ensure that they are suitable for AI analysis. AI models, especially LLMs, are trained in neurolinguistic programming to understand and interpret human language. In the context of TCM, this involves training models to recognize and analyze specific terminology that is used in patient inquiries. Afterward, AI models must be trained to understand the context in which TCM terms are used. This includes recognizing relationships between different symptoms and their implications with regard to overall health according to TCM principles. However, TCM is practiced worldwide, and patient inquiries may also be in various languages or dialects. AI models need to be trained on diverse data sets to ensure that they can handle different languages and cultural interpretations of TCM terms. A significant amount of labeled data and expert input are subsequently required for validation. Collaborations with TCM practitioners to annotate and validate data can improve the model’s accuracy. In summary, although AI with LLMs shows significant promise for managing EHRs, TCM inquiry demonstrates a unique knowledge system. The fine-tuning of LLMs is essential for transforming these general-purpose models into specialized models that are adept at handling TCM EHRs [18]. Future efforts should entail constructing a knowledge system for TCM diagnosis. It will then be necessary to fine-tune LLMs for TCM diagnosis based on existing LLM data models, thus providing AI tools for case analysis in TCM diagnosis. The integration of AI into the TCM inquiry process is a complex task that requires the careful consideration of unique aspects of TCM terminology and practice. With the right approach, including ongoing research and collaboration with TCM experts, AI can be effectively used to analyze patient data and enhance the diagnostic process in TCM.

### Palpation

Pulse diagnosis is one of the 4 main pillars of TCM assessment. By palpating the pulse at 3 specific positions on the wrists (“cun,” “guan,” and “chi”), practitioners can gain a comprehensive understanding of a person’s overall health and the state of specific organs. TCM pulse diagnosis consists of approximately 29 different pulse types that encompass a range of descriptors, including floating pulses and scattered pulses [19]. The intersection of pulse diagnosis and AI presents 2 main challenges. TCM pulse detection has historically relied on manually palpating the arteries beneath the skin to detect the pulse, thus lacking objective standards. In the process of AI-driven traditional Chinese pulse diagnosis, 2 critical issues need to be addressed: the development of pulse measurement devices and the standardization of pulse detection data. Lan et al [20] created a sensing device that features a multipoint sensor to measure pulse. Due to the complexity of pulse detection, previous methods that have primarily relied on multipoint sensors have only offered a limited scope of information. The development of pulse measurement devices has led to significant technological advances in recent years, and these advances are mainly reflected in innovations in sensor technology and the application of AI algorithms. Photovoltaic volumetric pulse wave sensors, which are based on photoplethysmography, are among the most common types of sensors used in pulse measurement devices. Pulse waves are measured by detecting the flow of blood in the microvasculature to obtain physiological parameters such as heart rate [21]. Photoplethysmographic sensors, such as smartwatches and fitness trackers, are widely used in consumer electronics. Some devices use pressure sensors to measure pulse waves, especially in continuous blood pressure monitoring. These sensors are often embedded in wearable devices that can monitor changes in blood pressure over time. To address the challenge of normalizing pulse data, AI algorithms preprocess the data before analysis, including filtering, denoising, and normalization, to ensure data quality. AI technology that is currently under development is working to improve the cross-device compatibility of algorithms so that data from devices from different manufacturers and models can be consistently analyzed, thus promoting data standardization and interoperability. The standardization of TCM pulse diagnosis is key to promoting the use of AI technology in TCM pulse measurements. It is necessary to establish unified pulse data and diagnostic standards along with integrating more diagnostic methods such as tongue diagnosis and diagnostic observation, from which we can develop an integrated TCM diagnostic platform and improve the comprehensiveness and accuracy of diagnosis.

In the future, more powerful multipoint sensing devices and multimodal detection devices will be needed to comprehensively examine pulse data and achieve better quantification. A challenge still remains in determining whether pulse data from these detectors can adequately reflect the characteristics of pulse diagnosis and in improving the classification of pulse patterns. To address the challenge of enhancing the precision of AI in interpreting pulse data for future research and development, noncontact pulse measurement techniques have demonstrated significant advancements. These methods eliminate the need for physical contact with the patient, which is particularly crucial for monitoring in unique or sensitive situations. For instance, the polarized multispectral imaging technique for noncontact heart rate measurement has refined the accuracy of data acquisition by pioneering new methods for extracting pulse waves from the palm [22]. This innovation contributes to the establishment of a standardized framework for pulse data, thus facilitating seamless data sharing and comparisons across various devices and systems. However, the attainment of high-quality data hinges on precise labeling, which is a process that can be both labor intensive and costly. In the context of electrocardiogram data annotation, the requirement for specialized physicians introduces variability, in which different medical professionals may offer conflicting assessments. This reality compounds the complexity and challenges associated with data preprocessing. To overcome these obstacles, it is imperative to refine data annotation protocols and invest in the development of more efficient and accurate labeling tools. By doing so, we can ensure that AI systems are trained on the most reliable data, thereby improving their diagnostic capabilities and contributing to the advancement of AI in health care. In summary, the development of detection methods and quantification of detection-based data are bottlenecks in the process of AI-driven pulse diagnosis.

### AI-Powered Tuina Massage Robot

Tuina massage (also known as Chinese medical massage) is a traditional hands-on manipulation treatment that is guided by the principles of TCM. It is widely used to treat various ailments, such as knee osteoarthritis, chronic neck pain, and insomnia [23-25]. The tuina massage serves 3 primary functions: facilitating the circulation of meridians, harmonizing qi and blood circulation, and augmenting the immune system [26-28]. These functions are essential for disease prevention and treatment and overall well-being. The integration of AI into tuina massage therapy is in its early stages. Efforts are underway to develop highly intelligent massage equipment and robotics based on TCM tuina to enhance its effectiveness, with a focus on improving the comfort, intelligence, and safety of massage robots [29]. Vibration and percussion are the 2 main types of tuina massage robotics. These devices offer acupressure techniques; however, manual massage from experienced physiotherapists provides additional popular movements, such as light stroking, stretching, and advanced kneading techniques, that machines cannot replicate. Therefore, the development of massage robots is a significant research focus for greater health care demands. Challenges mainly exist in their control, structure, and path planning; however, ongoing efforts aim to optimize their design and functionality. For example, the incorporation of a series-parallel hybrid structure may enhance flexibility while maintaining stiffness and precision [30]. Future research should focus on ergonomics to design high-performance massage robots that integrate advanced AI technologies for better control, sensing, and essential functions.

In current TCM tuina practice, the Expert Manipulative Massage Automation (EMMA) electronic massager, which was developed by AiTreat Pte Ltd in Singapore, is widely used. To deliver precise and effective massage based on muscle feedback, EMMA uses advanced sensor-based technology to identify focus points and adjust pressure levels. By detecting stiffness and resistance, EMMA can pinpoint muscle knots and tension points, thus applying varying pressure levels based on feedback and user preferences. In addition, EMMA incorporates Internet of Things technology for remote control, programming, and updates, thus enhancing its functionality in “green” Internet of Things applications. In EMMA technology, machine learning algorithms (especially convolutional neural networks in deep learning) are used to identify and analyze muscle tension patterns, acupuncture point locations, and physiological responses of patients. Through training, these algorithms are able to identify specific treatment points from sensor data to provide a customized massage solution for the patient. This pattern recognition capability allows the robotic massage therapist to pinpoint the area to be treated, thus mimicking the diagnostic process of an experienced massage therapist. The robotic masseur is able to adjust the intensity and speed of the massage based on real-time feedback from the patient. For example, if the sensors detect that a patient is experiencing discomfort at a certain pressure level, then the AI system can immediately adjust the intensity to ensure the comfort and effectiveness of the treatment. Through its advanced data analytics and learning capabilities, the AI application in EMMA technology is able to accurately identify treatment points and adjust massage intensity based on the patient’s real-time feedback. The EMMA massager has garnered high levels of acceptability and satisfaction among healthy volunteers, thus demonstrating its feasibility [31]. Nonetheless, research on massage robots still faces challenges, particularly regarding their clinical effectiveness. In addition, massage robots are categorized as class-I medical devices that do not require Food and Drug Administration approval for marketing in the United States [32]. Traditional medical device classification focuses on physical and biological characteristics, whereas the functionality of AI devices relies more on software and algorithms. Therefore, new classification criteria need to be developed that consider the specificities and potential risks of AI technologies. In the future, there will be a need for more standardized regulations to oversee research on massage robots [33]. Medical devices process and analyze large amounts of patient data, which requires regulations to include stringent requirements for data security and privacy protection. Medical device regulations need to incorporate specifications for data collection, storage, processing, and transmission to ensure the security and confidentiality of patients’ information, including the validation and clinical testing of AI algorithms. The use of AI medical devices involves the collection and analysis of large amounts of personal health information, which can threaten patients’ privacy. Regulations must ensure that the collection and use of patient information comply with privacy protection standards to prevent unauthorized access and data breaches. This will entail validating the functionality and therapeutic efficacy of medical devices to guarantee their safety and efficacy for users.

We propose the following perspective on the development of tuina robots. First, the overall stability of the tuina technique involves the stability of variable mechanical parameters and resulting morphological changes in mechanical effects during technique operation. These factors include mechanical characteristics such as force; speed; frequency; displacement; and kinematic features such as limb range of motion, joint angles, and overall movement amplitude. For example, the dexterity of the robotic arm is key to achieving an accurate simulation of a human masseur’s maneuvers; however, it requires sophisticated mechanical design, including joint flexibility, end-effector versatility, and overall structural stability. The robot arm’s control system also needs to process large amounts of data and make decisions in real time. This includes trajectory planning, motion control, and complex algorithms for force and position control. To ensure safety, collision detection and response mechanisms also need to be implemented. Consequently, tuina has significant limitations and subjectivity, thus making it difficult to objectively quantify and accurately assess efficacy. AI offers unique advantages in addressing this issue, which is primarily manifested in the digitization of tuina techniques (ie, the development of precision and flexibility in massage robots). Addressing the accuracy of the tuina technique is a prominent issue that may require more diverse AI algorithms to digitize massage techniques and analyze the clinical effects of different massage methods. The accuracy of Chinese massage largely depends on the precise positioning of acupoints. Researchers are developing a human body model based on the mechanism of “bone degree and minutes” in Chinese medicine, which realizes the calculation of 3D coordinate values of acupoints through robotics as well as identifying and tracking human body features by using such sensor technologies as depth cameras, thus realizing the precise positioning of acupoints. Second, another advantage of AI includes personalized health care services. The personalized parameter settings of massage robots are core parameters for future tuina robots. There are significant differences in individuals’ sensitivity and tolerance to pressure. The comfort and pain thresholds of people can vary, thus significantly affecting their experience with massage robots. AI can analyze the user’s physical condition, health data, and personal preferences to design a personalized massage program. Through the integration of advanced intelligent sensors, massage robots can monitor users’ physiological responses, such as muscle tension and body temperature, in real time. According to these data, massage robots can adjust their massage strength, speed, and focus area to overcome the “subhealth pain problem” of accurate positioning and efficient massage. At present, there are few studies on the clinical effectiveness of AI nudging robots, and patient self-reported changes in pain level, duration of pain relief, reduction in drug dependence, and objective measures of mobility (such as gait analysis) will be important indicators for evaluating their effectiveness in the future. When considering factors such as safety and comfort, AI data recording and analysis can also be used to measure the clinical efficacy of tuina robots.

## AI-Directed Acupuncture Manipulation

Acupuncture, which is a therapeutic technique in TCM that has been practiced for thousands of years, has gained widespread global acceptance and demonstrated significant efficacy for various chronic diseases, particularly pain-related conditions. This therapeutic approach involves stimulating specific areas, known as acupoints, on the patient’s body, thus eliciting sensations such as soreness, numbness, fullness, or heaviness, which is commonly referred to as “De Qi” or achieving qi [34]. Due to the inherent subjectivity and reliance on experience in traditional acupuncture practices, there is growing interest in parameter-based electroacupuncture to address these limitations [35]. By setting different parameters using an electroacupuncture device, clinical efficacy can be enhanced, thus facilitating further research. However, the efficacy of acupuncture is still not universally recognized [36], possibly for 2 main reasons. First, the inadequate design and implementation of past clinical research methods have led to a lack of clinical evidence. Second, the mechanism of acupuncture remains unclear, thus necessitating more high-quality evidence to elucidate its biological mechanisms for informed clinical decision-making [37]. The integration of AI and acupuncture shows great potential for substantially improving the precision of acupuncture prescriptions and treatment techniques. A bibliometric study by Zhou et al [38] demonstrated substantial progress in AI research within the acupuncture field over the past 2 decades, with significant contributions from the United States and China. However, the application of AI in acupuncture lacks a clear framework, with a scarcity of systematic research and a lack of organization of relevant technologies and application approaches.

Given the unique characteristics of AI and the importance of data mining in clinical acupuncture practice and manipulation, further research is needed [39]. Clinical trials are costly and limited, and most articles that analyze the safety and efficacy of acupuncture are of low quality and lack comprehensive analyses. There is still a lack of standardized acupuncture point selection protocols for many diseases [40]. Therefore, future efforts should focus on standardizing TCM while improving the quality of randomized controlled trials on acupuncture to obtain more and higher-quality clinical data, thus providing a foundation for AI-based clinical data mining. AI can analyze a patient’s symptoms, signs, and physiological data and compare them to a large body of medical knowledge. Through machine learning and pattern recognition algorithms, AI can help clinicians interpret diagnostic data and provide potential pathological patterns or disease classifications that can help acupuncturists in developing treatment strategies. AI can then be used to analyze large amounts of clinical data and research the literature to determine the most effective point selection for a particular condition or disease situation. A recent study “linked” original studies and 332 systematic evaluations of evidence in 20 disease areas by using AI analysis techniques to comprehensively improve clinical evidence for acupuncture therapy in the Epistemonikos database, which constructed a total of 77 evidence matrices [41]. This will facilitate the development of a machine learning framework to predict the efficacy of acupuncture and patient prognosis. Acupuncture manipulation techniques are crucial components of acupuncture therapy, and their efficacy is paramount [42]. However, the determination of the optimal stimulation intensity in clinical research is often challenging because of technique selection, treatment duration, needling speed, and force [43-45]. Therefore, the quantification and standardization of acupuncture manipulation, such as needle insertion force, duration, and direction, are essential for achieving clinical efficacy and AI-guided acupuncture manipulation. In response to the problems in standardizing operation techniques, AI technologies, especially machine learning models and sensor technologies, are being used to capture and analyze the nuances of manual needling operations. Acupuncture robots that are currently under development can accurately gauge the location of acupuncture points by measuring a person’s height and sebum thickness and use ultrasound sensors to control the depth and speed of needling. These sensors and machine learning models are able to identify key parameters such as the needling force, speed, and angle to ensure the standardization and consistency of treatment. The application of sensor technology in acupuncture focuses on the precise control and measurement of the depth, force, and speed of needling. This robot uses an ultrasound sensor to control the depth and speed of needling. By emitting ultrasonic waves and receiving their echoes, the ultrasonic sensor can accurately measure the distance between the tip of the needle and the surface of the tissue to ensure that the depth of the needles is appropriate and avoid unnecessary injury to the patient. Through built-in mechanical sensors, the robot can also automatically adjust the needle insertion process according to changes in needle insertion resistance to ensure safe needle insertion.

The standardization of acupuncture manipulation forms the basis of the use of AI in acupuncture. With AI technology, we propose three different ways to help standardize acupuncture: (1) imaging recognition–based standardization of acupuncture practitioners’ techniques, (2) analysis of parameters derived from acupuncture practitioners’ lifting and thrusting techniques using neural network image analysis systems, and (3) extraction of spatiotemporal features from video images of acupuncture operations by using computer vision technology [46]. In addition, a hybrid model that combines 3D convolutional neural networks and neural networks is used to recognize and classify dynamic hand gestures in acupuncture operation videos, thus enabling quantitative analyses and technical inheritance research for various techniques. Another approach involves recording acupuncture practitioners’ movements and mechanical parameters during acupuncture procedures by using 3-axis posture sensors. Davis et al [47] developed force and motion sensor technology (acusensors) to quantify the linear and rotational movements of acupuncture needles and the force and torque that are generated during manual needle manipulation. A standardized TCM acupuncture manipulation database was established for the quantification of motion and force patterns. These data serve as a crucial tool for future AI applications in acupuncture. Finally, acupuncture parameters based on other electrophysiological signals have been recorded, thus showing significant differences in electrophysiological signals between acupuncture points and nearby nonacupuncture points and highlighting the electrical specificity of acupoints [48,49]. This finding serves as compelling evidence for TCM theory and provides parameters for the standardization of acupuncture stimulation. In addition, collaboration between AI experts, acupuncturists, and biomedical engineers is essential for developing and improving acupuncture-related technologies. The data analysis and intelligent algorithms that are provided by AI experts can help acupuncturists better understand treatment effects and optimize treatment plans. The clinical experience and theoretical knowledge of acupuncturists can guide AI experts in developing intelligent systems that better meet clinical needs. Moreover, there are some prominent conditions or events existing outside of normal circumstances that exist beyond the abilities of AI. In such cases, acupuncturists can make judgments based on their own experience and knowledge. Technical support from biomedical engineers subsequently ensures that these intelligent systems can be effectively applied in practice. Close collaboration among the 3 factors is the key to promoting technological innovation in acupuncture, improving treatment outcomes, and standardizing and popularizing acupuncture techniques. Through this interdisciplinary collaboration, modern technology can be better used to enhance the value and impact of traditional acupuncture medicine. AI technology can be used to simulate acupuncture operations and provide support for learning and training. For example, through virtual reality and augmented reality technologies, AI can create simulated acupuncture treatment scenarios that allow learners to practice acupuncture techniques and decision-making processes in a virtual environment. This approach improves learning efficiency and reduces risks in actual practice.

## Outlook

**Figure 1:**
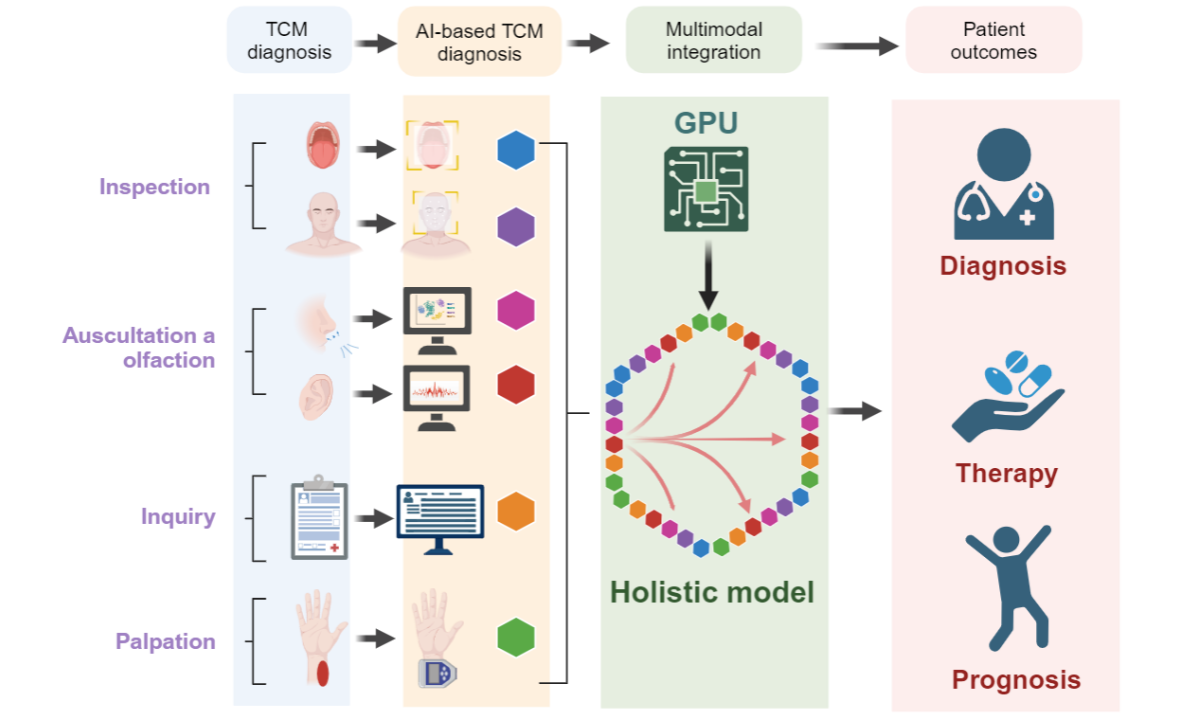
Overview of artificial intelligence (AI) development strategies based on traditional Chinese medicine (TCM) diagnosis. The acquisition and standardization of unimodal data through TCM diagnostic techniques is followed by the integration of multimodal data using a comprehensive model. This approach aids in enhancing predictions and supports TCM diagnoses for treatment and prognosis. GPU: graphics processing unit.

The future is promising for the integration of AI into TCM diagnosis (Figure 1). The core of TCM diagnosis involves 4 fundamental methods: inspection, auscultation and olfaction, inquiry, and palpation. First, through inspection, we can explore the application of AI in facial and tongue diagnosis. By leveraging image processing techniques, we anticipate groundbreaking innovations in these areas. Due to the low contrast and noisy nature of ultrasound images, which require extensive knowledge of tongue structure and ultrasound data interpretation, it can be challenging for medical professionals to accurately diagnose various conditions. To overcome these challenges, researchers have proposed new deep neural networks, referred to as BowNet models [50]. These models leverage the global prediction capabilities of encoder-decoder fully convolutional neural networks and the high-resolution extraction features of dilated convolutions. The models are designed to automatically extract and track the tongue contour in real-time ultrasound data. By providing more accurate and objective tongue contour tracking, AI can assist TCM practitioners in making more informed diagnoses and treatment decisions. However, the digitization of valuable empirical data from TCM facial and tongue diagnosis remains a challenge, although unsupervised machine learning may provide a solution. First, addressing the challenge of digitizing TCM experience data often requires seamless collaboration among diverse teams, which encompasses TCM practitioners, AI experts, and health care institutions. This initiative involves partnering with medical facilities to meticulously gather and curate a rich array of TCM clinical case data, thus encompassing detailed patient complaints, symptomatic expressions, tongue diagnoses, and pulse assessments. Subsequently, synergy between TCM professionals and AI researchers has extended to the intricate task of knowledge structuring. The translation of the profound insights of TCM theories, herbal formulas, and medicinal properties into a web-based knowledge graph can greatly enhance the accessibility and utility of this ancient medical wisdom. This graph facilitates a nuanced understanding of intricate relationships within TCM, underpins the development of intelligent recommendation systems, and assists in informed decision-making within the realm of TCM. Through these collaborative efforts, the rich tapestry of TCM experience data is meticulously incorporated into accessible, digital formats, thereby bridging the gap between ancient wisdom and modern technological advancements. These initiatives have propelled the modernization and globalization of TCM and significantly contributed to the broader landscape of health care innovation. Second, TCM diagnosis through auscultation and olfaction provides new avenues for development. We can develop specialized AI tools for auditory and olfactory systems that can complement TCM diagnosis. Third, the analysis of inquiry in TCM diagnosis presents an exciting frontier. There has been significant progress in Western medicine in this area with regard to LLMs, and TCM EHRs contain unique knowledge graphs that are specific to TCM diagnosis. Therefore, fine-tuning is necessary for the analysis of TCM EHRs. Fourth, pulse diagnosis is a cornerstone of TCM diagnosis and requires the development of more advanced pulse detection tools. By obtaining substantial pulse data, we can standardize objective pulse analysis. By leveraging machine learning and classification techniques, we can align these data with TCM pulse patterns, thus ultimately achieving AI-driven pulse diagnosis. Quantifiable metrics or benchmarks are instrumental in ensuring the high performance of AI systems, establishing unambiguous objectives, and measuring advancements in the integration of AI technologies. For example, metrics such as diagnostic accuracy and generalization capability can assess AI systems’ proficiency in managing patient data across varying regions, age groups, and sexes, thereby ensuring their efficacy across a wide array of populations. By juxtaposing outcomes that are generated by AI systems with diagnoses that are rendered by seasoned TCM experts, the precision of AI systems in pinpointing specific conditions can be quantified. This quantification is facilitated through the computation of statistical indicators such as sensitivity (true positive rate), specificity (true negative rate), precision, and the *F*_1_-score. These benchmarks serve as a foundation for the continuous refinement of AI systems, thus ensuring their optimal integration and application within the sphere of TCM. These directions of development have the potential to revolutionize TCM diagnosis, thus enhancing its accuracy and efficiency.

## Challenges

**Figure 2:**
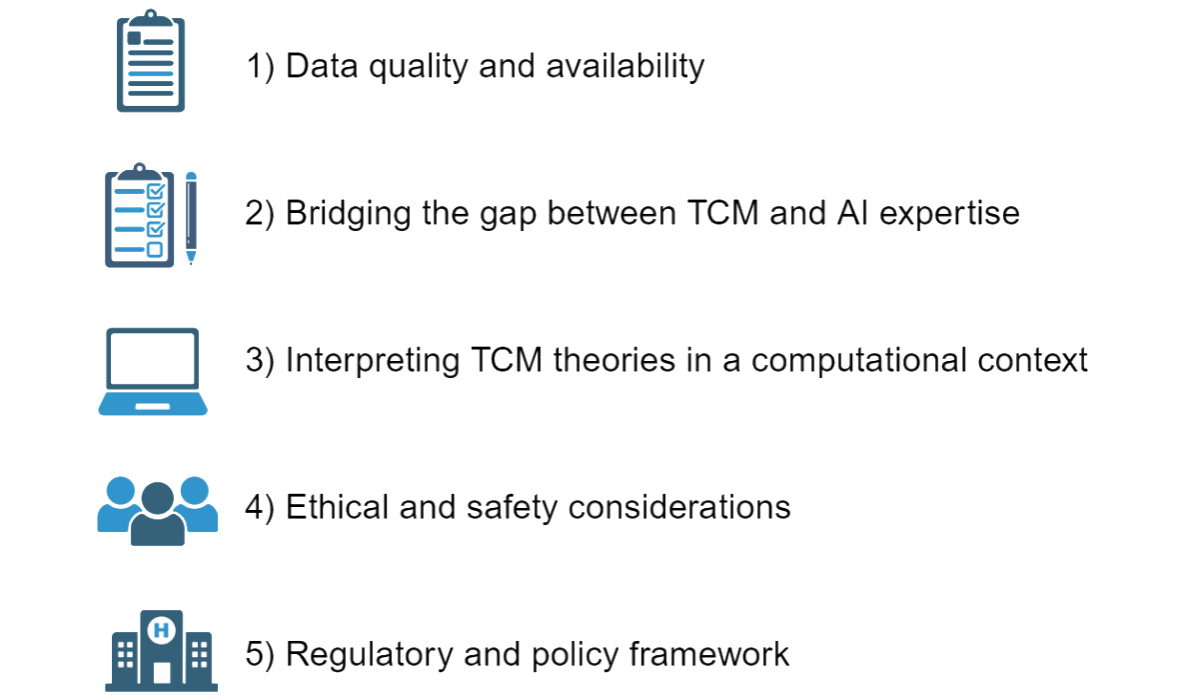
Summary of the challenges of integrating artificial intelligence (AI) into traditional Chinese medicine (TCM) diagnosis.

There are unique challenges to the use of AI in TCM (Figure 2). First, regarding data quality and availability, the successful implementation of AI in TCM relies on access to reliable and standardized data sets. High-quality data can potentially promote clinical diagnosis and treatment in precision TCM. However, data collection and digitization efforts in TCM can be challenging, and the quality and interoperability of existing data sets may vary. Varied interpretations of identical conditions among TCM practitioners can result in divergent diagnostic criteria and terminological applications. Such disparities, coupled with the potential for errors and biases in manually entered data, can significantly impact the learning efficacy of AI systems. Consequently, the establishment of standardized TCM diagnostic criteria and terminology glossaries, in addition to the implementation of uniform data entry protocols for TCM practitioners, is essential for mitigating interpractitioner discrepancies. Furthermore, the use of advanced automated data collection techniques, including image recognition and natural language processing, is instrumental in enhancing the quality and precision of the collected data. These measures collectively contribute to the refinement of the analytical capabilities of AI within the realm of TCM, thus ensuring a more accurate and reliable diagnostic process. Second, to bridge the gap between TCM and AI expertise, the integration of AI technologies into TCM requires collaboration and communication among TCM practitioners and AI experts. Bridging the gap between these domains is crucial for developing AI algorithms that align with TCM principles and meet specific clinical needs. Measures could be taken to promote collaboration between TCM organizations and technology companies, as well as higher-education institutions, to facilitate the development of AI-driven TCM diagnostic and therapeutic tools. These collaborations will foster innovation and create platforms for TCM students and practitioners to perform internships in the technology industry. In addition, the provision of scholarships and research grants is critical for incentivizing and sustaining interdisciplinary scholarship. By allowing students and researchers to delve into the convergence of TCM and AI, we can accelerate the digitization of TCM knowledge. Third, TCM theories must be interpreted in a computational context. TCM theories are often complex and based on holistic and individualized perspectives. The translation of this knowledge into AI algorithms and computational models is a significant challenge that requires careful consideration of cultural, philosophical, and theoretical aspects. Many concepts in Chinese medicine, such as qi, yin and yang, and the 5 elements, are abstract and ambiguous, and it is difficult to describe these concepts in precise mathematical language; therefore, ambiguous logic can be used to address ambiguous concepts in TCM, and Bayesian networks can be used to simulate causality and uncertainty in the theory of Chinese medicine. Fourth, there are notable ethical and safety considerations regarding this scenario, as with any implementation of AI in health care [51]. Ensuring patient privacy, data security, and transparency in algorithm decision-making is essential for building trust and ethical practices in AI-supported TCM. Informed patient consent must be obtained before collecting and using patient data. This includes a full explanation of the purpose of data collection, how the data will be used, how long they will be stored, and potential risks. To protect patients’ privacy, all the data sets that are used for machine learning should be anonymized by removing or encrypting any personally identifiable information. During the development and deployment of AI systems, ethical review committees need to be established to ethically review the design, implementation, and evaluation of AI systems to ensure that all the activities meet ethical standards. Fifth, the integration of AI into TCM necessitates clear regulatory frameworks and policies that govern its implementation, including issues related to data protection, algorithm validation, and clinical decision-making. Sixth, the function and efficacy of TCM are broadly accepted worldwide; however, the underlying mechanism has remained enigmatic, thus limiting people’s confidence in TCM and precision therapy that is learned by AI. In summary, the process of implementing and validating AI tools in a clinical setting requires careful planning and rigorous execution. Representative and actionable clinical environments are selected to develop pilot projects. These projects should focus on specific TCM diagnostic tasks, such as tongue analysis, pulse recognition, and symptom assessment. Clinical trials need to be designed and executed to evaluate the performance of AI tools in real clinical settings. This includes randomized controlled trials and prospective cohort studies to assess the impact of AI tools on patient outcomes. Finally, the results and experiences will be published and shared through academic journals and conferences to promote communication and learning within the industry.

## Conclusions

In conclusion, the integration of AI into TCM exhibits immense promise for improving diagnosis, including inspection, auscultation and olfaction, inquiry, and palpation. The successful integration of AI into TCM is evident through advancements in areas such as image analysis for tongue diagnosis, the development of intelligent tuina massage systems, and the application of machine learning to refine treatment protocols based on individual patient data. Addressing the challenges of data quality, the standardization of data sets, interdisciplinary collaboration, the interpretation of TCM theories, ethical considerations, and regulatory frameworks is crucial for the successful and responsible implementation of AI in TCM. By overcoming these challenges, we can leverage the power of AI to enhance patient care, personalize treatments, and advance our understanding of TCM. Moreover, we can develop more precise AI models that are tailored to TCM, thus creating a positive cycle of problem-solving and progress that ultimately leads to better patient care. By combining the wisdom of TCM with the power of AI technology, we can improve patient outcomes and promote the integration of TCM into modern health care systems. It is imperative to conduct more research into AI’s ability to decode complex diagnostic patterns that are inherent to TCM. The validation of AI-enhanced TCM treatment methods through clinical trials is essential to ensure their safety and efficacy, thus providing empirical support for their widespread adoption. As we advance the integration of AI into TCM, it is vital to uphold ethical standards that prioritize patient rights, cultural integrity, and data privacy. The responsible use of AI will ensure that technological advancements align with the principles and practices of TCM, thus safeguarding the well-being of patients and respecting the cultural significance of this ancient medical system. The fusion of AI with TCM has the potential to bridge traditional and modern medical practices, enrich global health, and foster cultural exchange. By integrating these 2 domains, we can create a more comprehensive health care system that is both innovative and respectful of historical practices.

Finally, a call to action is made to all stakeholders (practitioners, researchers, policy makers, and investors) to collaborate and support the integration of AI into TCM. Through collective efforts, we can harness AI to transform patient care, broaden our understanding of TCM on a global scale, and identify new horizons in health care that are both deeply rooted in tradition and boldly futuristic.

## Abbreviations

AI: artificial intelligence
EHR: electronic health record
EMMA: Expert Manipulative Massage Automation
LLM: large language model
TCM: traditional Chinese medicine
